# Embryo Density and Its Neutrality in Day-3 Embryo Development: A Retrospective Cohort Study

**DOI:** 10.1155/2022/6368678

**Published:** 2022-03-12

**Authors:** Cheng Shi, Tie Cheng Sun, Shi Hui Meng, Ping Wang, Rong Liang, Sheng Nan Duan, Hong Jing Han, Ya Nan Liu, Huan Shen, Xi Chen

**Affiliations:** ^1^Reproductive Medical Center, Peking University People's Hospital, Peking University, Beijing 100044, China; ^2^Department of Obstetrics and Gynecology, Peking University International Hospital, Beijing 102206, China; ^3^Department of Obstetrics and Gynecology, Second Affiliated Hospital of Soochow University, Suzhou 215006, China

## Abstract

**Introduction:**

Many studies have shown that embryo density has an impact on day-3 embryo-developmental outcomes; however, embryo density remains controversial in clinical practice. We aimed to evaluate the association between embryo density and day-3 embryo-developmental outcomes in real world with the largest sample size.

**Methods:**

In 2018, we identified 10941 day-3 embryos from all female patients (*n* = 1568) in the study. The embryos were allocated to three embryonic densities: 30 *μ*l/embryo (individual culture), 15 *μ*l/embryo, and 10 *μ*l/embryo (group culture). The primary outcomes were cleaving speed, quality, and proportion of successful implantations. The generalized estimate equation (GEE) model was used both in the univariate analysis and multivariable logistic regression analyses to investigate the relationship between embryo density and embryo-developmental outcomes.

**Results:**

There were 3064, 5695, and 2182 embryos in the 30 *μ*l/embryo group, 15 *μ*l/embryo group, and 10 *μ*l/embryo group, respectively. The proportions of 7–10 cell embryos were 57.2%, 56.1%, and 58.3% in three densities with no statistical significance (*P*=0.37), respectively. The proportions of morphologically good embryos were 20%, 20.3%, and 20% in three densities with no statistical significance (*P*=0.85), respectively. Proportions of implanted embryos were 37.7%, 37.1%, and 27.8% with no statistical significance (*P*=0.36), respectively. After adjustment for confounders, which were significant in the univariate analysis, the embryo density was still not associated with day-3 embryo-cleaving speed, day-3 embryo quality, and day-3 embryo-implanting potential (all *P* > 0.05).

**Conclusion:**

In a 30 *μ*l microdrop, the culturing embryos with embryo densities of 15, 10, and 30 *μ*l/embryo (from zygotes to day 3) had similar developmental outcomes. The embryo density had no impact on day-3 embryo development.

## 1. Introduction

In the practice of human in vitro fertilization and embryo transfer (IVF-ET), optimization of the in vitro environment involves improvement of the culture-media formulations and the provision of other physical, culturing conditions. Individual culture, group culture, and different embryo density cultures consist of culturing conditions. Group culture is remarkably superior to individual culture [1–5] in the developmental outcome of mammalian embryos and human blastocysts [2, 5–7]. However, group culture has not been the common culture method, through IVF-ET laboratories worldwide [8]. Still, the need of individual culture arises for the improvement of noninvasive embryo-screening techniques. The blastocysts' developmental outcome is mainly affected by the quality of day-3 embryos; therefore, it is critical to clarify the optimal, culture method from zygotes to day-3 embryos, of which there is still no consensus. It has been known that some demographical and cycle characteristics' confounding factors, such as maternal age [9], insemination type [10], and number of retrieved oocytes [11], can directly affect day-3 embryos' developmental outcome. Therefore, it is important to adjust these confounders when investigating the relationship between the culture method and the embryo-developmental outcome. However, previous similar studies seldom adjusted these confounding factors.

In this study, the relationship between the embryo density and the developmental outcome of day-3 embryos was investigated, with the following confounders adjusted: the maternal age, paternal age, antral follicles, level of anti-Müllerian hormone (AMH), type of infertility, controlled ovarian stimulation (COS) protocol, length of stimulation, number of retrieved oocytes, and insemination type. The embryos were cultured with different embryo densities in group culture or individual culture, and the developmental outcomes of day-3 embryos were analyzed and compared after adjusting confounders.

## 2. Materials and Methods

### 2.1. Patients Selection and Study Design

In 2018, we retrospectively collected 10941 embryos from all 1568 female patients treated in the Department of Reproductive Medicine Center, Peking University People's Hospital (Peking, China). The patients who intended to undergo IVF-ET were included. Exclusion criteria were as follows: patients who got no oocyte after controlled ovarian hyperstimulation and oocytes retrieval; patients who did not have normally fertilized zygotes (with two pronuclei 16–18 h after insemination); and patients who only had degenerated oocytes. There was no preimplantation genetic testing [PGT] or donor cycles in our center and embryos were not individually cultured according to our laboratory's protocol. Every patient was informed in detail about the IVF treatment procedures and risks, and the consent was obtained. The participants' informed consent was waived because of the retrospective cohort study. The study was approved by the Ethics Review Board of the Peking University People's Hospital (2018PHB061-01).

### 2.2. Ovarian Stimulation

Different COS regimens (i.e., the long, antagonist, and others (minimal-stimulation, natural, luteal phase stimulation, and progestin-primed stimulation)) were used according to the patients' characteristics and responses during previous IVF cycles or ovulation induction. When the diameter of at least one leading follicle was 18 mm, ovulation was induced with 5000–10,000 IU of human chorionic gonadotropin (hCG; Choragon, Ferring, Switzerland), alone or in combination with 0.2 mg triptorelin acetate (Ferring). Transvaginal ultrasound-guided oocyte retrieval was performed at 36 h after hCG administration.

### 2.3. IVF and Embryo Culture

After retrieval, the oocytes were incubated in a fertilization medium in a 60 mm IVF dish (353653; Falcon, Franklin Lakes, NJ, USA). Meanwhile, fresh sperm samples were collected from the male partners by masturbation. After the 80/40% density gradient centrifugation (Sage, USA), the sperm samples were kept in a 37°C, 6% CO_2_ incubator until insemination. Based on the sperm sample's quality, oocyte count, and previous IVF cycle performance, the oocytes were inseminated by conventional IVF or introcytoplasmic sperm injection (ICSI) at 40 h after hCG injection. Conventional IVF was performed by transferring the prepared sperm sample into the dish where the oocytes were incubated. For ICSI, the oocytes' cumulus cells were removed at 1 h after retrieval by gently pipetting, following a short, enzymatic digestion in hyaluronidase (Sage). The denuded oocytes were incubated in the cleavage medium and were then subjected to sperm injection in a gamete manipulation buffer at 40 h after hCG injection; the oocytes were immediately transferred to the culture dish in cleavage medium post-ICSI.

The embryo culture was performed in a COOK incubator with mixed gas of 5% O_2_, 6% CO_2_, and 89% N_2_. The embryos were cultivated in 35 mm polystyrene dishes (150255; Nunc, USA). Six culturing and three washing drops (each 30 *μ*l) were separately placed onto the circle and center of the dish's bottom the day before oocyte retrieval. In detail, culture medium (10 *μ*l; G1-PLUS, Vitrolife, Sweden) was directly pipetted directly onto the dish, overlaid by oil (3.5 ml; Vitrolife), and then refilled with another culture medium (20 *μ*l). The dishes were immediately prepared at room temperature without airflow and then equilibrated overnight in a 37°C, 6% CO_2_ incubator before use. Generally, one to two dishes were prepared for each patient, with 9 drops in each dish.

The pronuclei were examined under a microscope at 16–18 h after insemination. Then, one, two, or three zygotes (with two normal pronuclei) were randomly and unselectively placed in a single microdrop of the culture dish for the ongoing culture. One or two embryos per drop were individually prepared. If there were still more embryos after all the drops were used, three embryos were then placed together into one drop. Zygotes with three or more pronuclei and degenerated oocytes were discarded. Zygotes with one pronucleus and unfertilized oocytes were subjected to group culture in different microdrops, which were not included in this study. On day 3, the embryo morphology was recorded based on the scoring system reported by Puissant et al. [12]. Briefly, the number and evenness of the blastomeres were analyzed, as well as the fragment percentages. Cleaved embryos with 7–10 equal or slightly unequal blastomeres and ≤15% fragments were considered as grade I. When the percentage of fragments was 16%–29% or the number of blastomeres did not meet the grade I standard, the embryos were considered as grade II. When the percentage of fragments was between 30% and 49%, the embryos were considered as grade III. Finally, when there were 50% fragments or the embryo development was retarded, the embryos were considered as grade IV. Embryos at grade I or II were selected with priority for transferring or freezing on day 3, and the others were group cultured in blastocyst medium for another 2 or 3 days.

### 2.4. Study Design and Data Collection

Demographic and treatment cycle information, such as maternal age, maternal body mass index (BMI), paternal age, number of antral follicles, anti-Mullerian hormone (AMH), infertility type, controlled ovarian stimulation (COS) protocol, length of stimulation, number of retrieved oocytes, and type of insemination, were collected from medical records. The study's target independent variable was embryo density, which was defined as microdrop volume divided by number of cultivated embryos. Generally, there were three different embryo densities in our IVF-ET embryo laboratory: 30 *μ*l/embryo (one embryo in a microdrop); 15 *μ*l/embryo (two embryos together in a microdrop), and 10 *μ*l/embryo (three embryos together in a microdrop). Primary outcomes were the cleaving speed, quality, and clinical result of day-3 embryos. Cleaving speed of day-3 embryos was defined as whether the embryos were cleaved or uncleaved to 7–10 cells on day 3. The quality of a day-3 embryo was indicated as a morphologically good embryo (MGE), which was defined as an embryo cleaved to having 7–10 equal or slightly unequal blastomeres and having ≤15% fragments. The clinical result was indicated by successful implantation, which was defined as a gestational sac in the cavity. All dependent variables were recorded as binary variables.

### 2.5. Statistical Analysis

Since data were not normally distributed, continuous variables were presented as mean ± standard or median (Q1, Q3). Categorical variables were expressed in frequency and percentage. Univariate analysis was employed to test the impact of each variable on our three outcomes. To find the real relationship between embryo density and the cleaving speed, quality, and clinical result of day-3 embryos, multivariable logistic regression analyses were employed. Each participant had several embryos, which were assigned to different embryo density groups. A generalized estimate equation (GEE) model, based on the patient's unique, medical record number, was used both in the univariate analysis and multivariable logistic regression analyses to avoid the repeated, measurement effect of participants' characteristics. In multivariable logistic regression analyses, we constructed three models: model 1, no confounders were adjusted; model 2, significantly different factors in the univariate analysis were adjusted; model 3, all the confounders were adjusted. To verify the results of multivariable logistic regression analyses based on embryo density as a categorical variable, we performed a sensitivity analysis by converting the embryo density into a continuous variable and calculating the *P* value for the trend. All data were analyzed by statistical product and service solutions (SPSS) 23.0 (IBM, NYU). *P* values less than 0.05 (two-sided) were considered statistically significant.

## 3. Results

### 3.1. Baseline Characteristics of Patients

**Figure 1:**
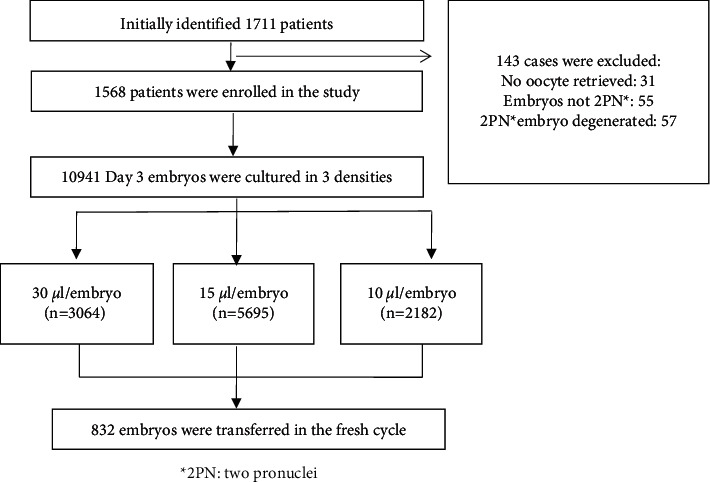
Flow chart of selection of 1568 patients with 10941 embryos.

A total of 1568 patients were enrolled, and 10941 embryos were cultured in the study (Figure 1). Patients' demographic and treatment cycle characteristics are shown in Table 1. Mean ages of the females and their husbands were 33 (30–36) and 35 (31–38), respectively. Female patients' body mass index (BMI) was 22.3 (20.3–25.4); they had 9 (5–14) antral follicles and the AMH levels of them were 2.88 ng/ml (1.50–5.23 ng/ml). Approximately 7.78% patients received a long and antagonist protocol, 49.7% were diagnosed with primary infertility, and 66.6% had their oocytes inseminated by conventional in vitro fertilization (IVF). Female patients underwent 10 (8–11) days' stimulation and had 10 (5–15) oocytes retrieved.

### 3.2. Cleaving Speed, Quality, and Clinical Results of Day-3 Embryos among Different Embryo Density Groups

A total of 10,941 embryos were assigned into three embryo density groups: 3064 embryos in group 1 (30 *μ*l/embryo), 5695 embryos in group 2 (15 *μ*l/embryo), and 2182 embryos in group 3 (10 *μ*l/embryo; Table 2). Approximately 57.2%, 56.1%, and 58.3% embryos were cleaved to 7–10 blastomeres on day 3 (named 7–10 cell embryos) in groups 1, 2, and 3.

Approximately 20%, 20.3%, and 20% embryos in groups 1, 2, and 3, respectively, were evaluated as morphologically good embryos (MGE) on day 3: 430 embryos in group 1, 348 in group 2, and 54 in group 3, which were transferred into the fresh, controlled ovarian stimulation cycle, separately resulting in successful implantation of 162, 159, and 15 embryos, respectively. None of the above outcomes had significant differences among the three embryo density groups.

### 3.3. Impact of Each Variable on Cleaving Speed, Quality, and Clinical Results of Day-3 Embryos from Univariate Analysis Based on GEE Model

**Figure 2:**
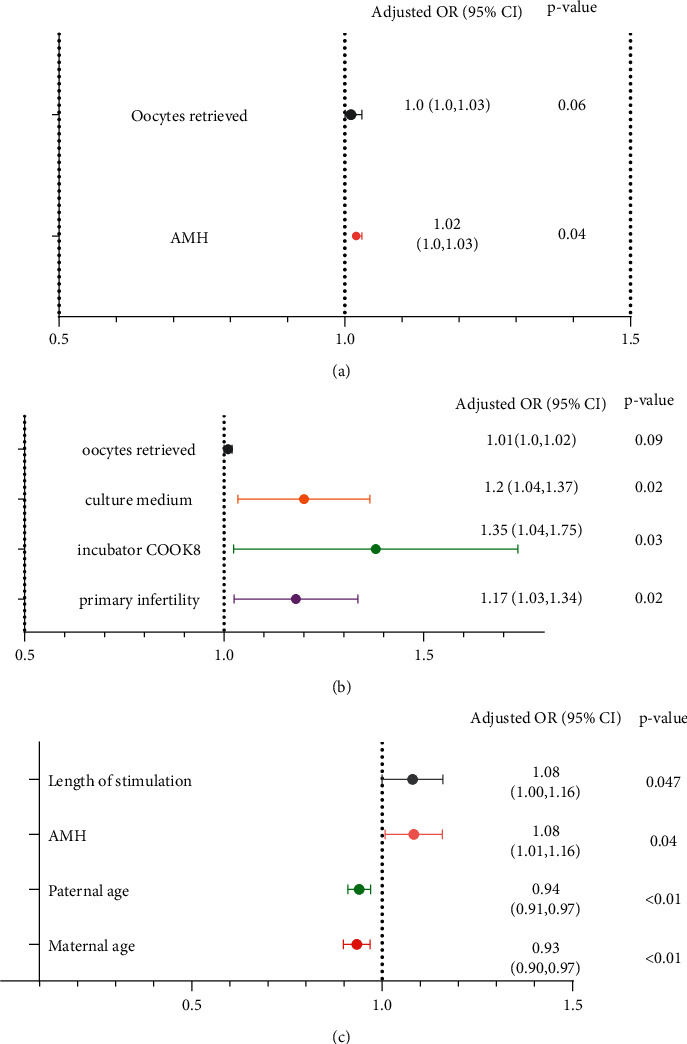
Univariate logistic regression analysis of cleaving speed, quality, and clinical results of day-3 embryos. (a) Variables associated with 7–10 cell embryos; (b) variables associated with MGE; (c) variables associated with successful implantation. AMH: anti-Mullerian hormone; MGE: morphological good embryos.

For the outcome of cleaving speed (recorded as “whether the embryo cleaved to 7–10 cells on day 3 or not,” a binary variable), only female patients' AMH had significant power. Along with the increased AMH levels of female participants, more embryos were cleaved to 7–10 cells on day 3 (OR = 1.02, 95% CI: 1.00–1.03, *P*=0.04). For the embryo-quality outcome (recorded as “whether the embryo was an MGE on day 3 or not,” a binary variable), primary infertility, incubator COOK8, and culture medium had significant power. Specifically, comparing to patients who had a secondary infertility, patients who had a primary infertility had more embryos evaluated as MGE on day 3 (OR = 1.17, 95% CI: 1.03–1.34, *P*=0.02). Comparing to embryos cultured in a COOK1 incubator, more embryos were cultured in a COOK8 incubator which were evaluated as MGE on day 3 (OR = 1.35, 95% CI: 1.04–1.75, *P*=0.03). Comparing to embryos cultured in a COOK medium, more embryos cultured in a Vitrolife medium were evaluated as MGE on day 3 (OR = 1.19, 95% CI: 1.04–1.37, *P*=0.02). For the outcome of the clinical results (recorded as “whether the transferred embryo implanted successfully or not,” a binary variable), maternal age, paternal age, AMH, and length of stimulation had significant power. Specifically, along with the increased age of female and male participants, less embryos were successfully implanted (OR = 0.93, 95% CI: 0.90–0.97, *P* < 0.01; OR = 0.94, 95% CI: 0.91–0.97, *P* < 0.01). In contrast, along with the increased AMH level of female participants and length of stimulation, more embryos were successfully implanted (OR = 1.08, 95% CI: 1.01–1.16, *P*=0.04; OR = 1.08, 95% CI: 1.00–1.16, *P*=0.047) (Table 3) (Figure 2).

### 3.4. Relationship between the Embryo Density, Cleaving Speed, Quality, and Clinical Results of Day-3 Embryos in Different, Multivariable Logistic Regression, GEE Models

**Figure 3:**
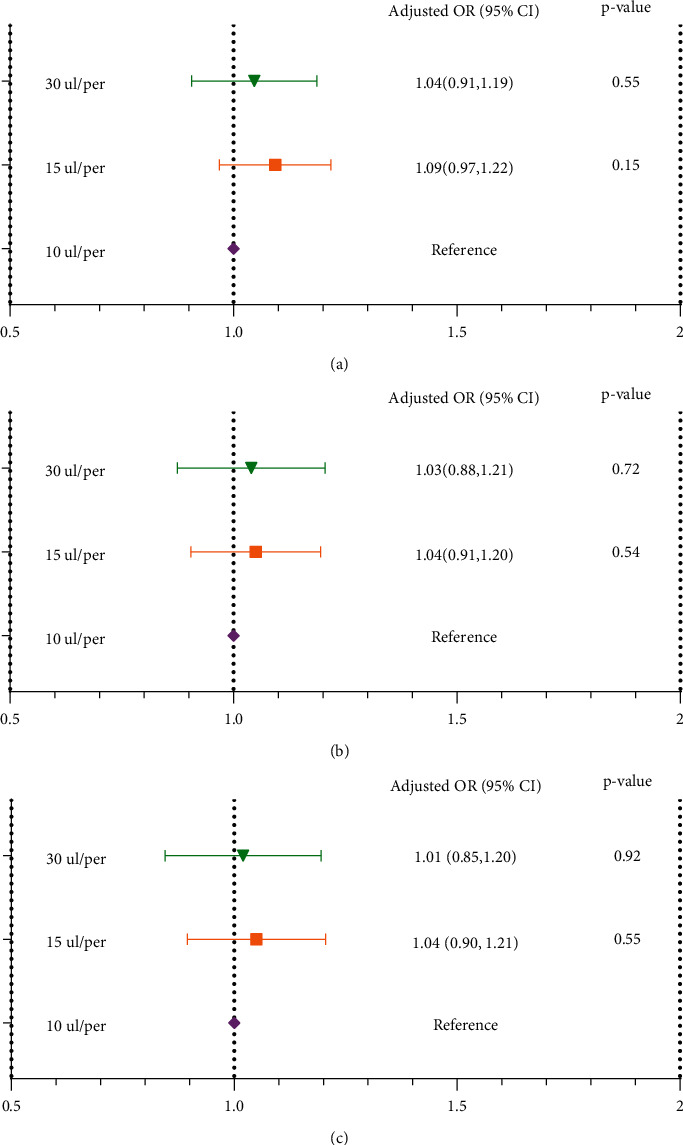
Relationship between embryo density and cleaving speed of day-3 embryos in different models. The analysis was performed by GEE (generalized estimate equation) model, subject ID = patient unique medical record number (independence). (a) Crude model: no factor was adjusted; (b) Model I: the number of retrieved oocytes and AMH were adjusted; (c) Model II: maternal age, paternal age, maternal BMI, type of infertility, type of insemination, COS protocol, AMH, the number of retrieved oocytes, length of stimulation, antral follicles, incubator, and culture medium were adjusted.

As shown in Figure 3, there was no adjustment in the crude model, and the embryo density had an insignificant effect on cleaving speed (group 30 ul/embryo: *P*=0.55, group 15 ul/embryo: *P*=0.15, group 10 ul/embryo: reference). The AMH and number of retrieved oocytes (significant risk factors in the univariate analysis) were adjusted in Mode I, and the embryo density still had an insignificant effect (group 30 ul/embryo: *P*=0.72, group 15 ul/embryo: *P*=0.54, group 3: reference). All confounders were adjusted in Model II, and the embryo density still had an insignificant effect (group 30 ul/embryo: *P*=0.92, group 15 ul/embryo: *P*=0.55, group 10 ul/embryo: reference).

**Figure 4:**
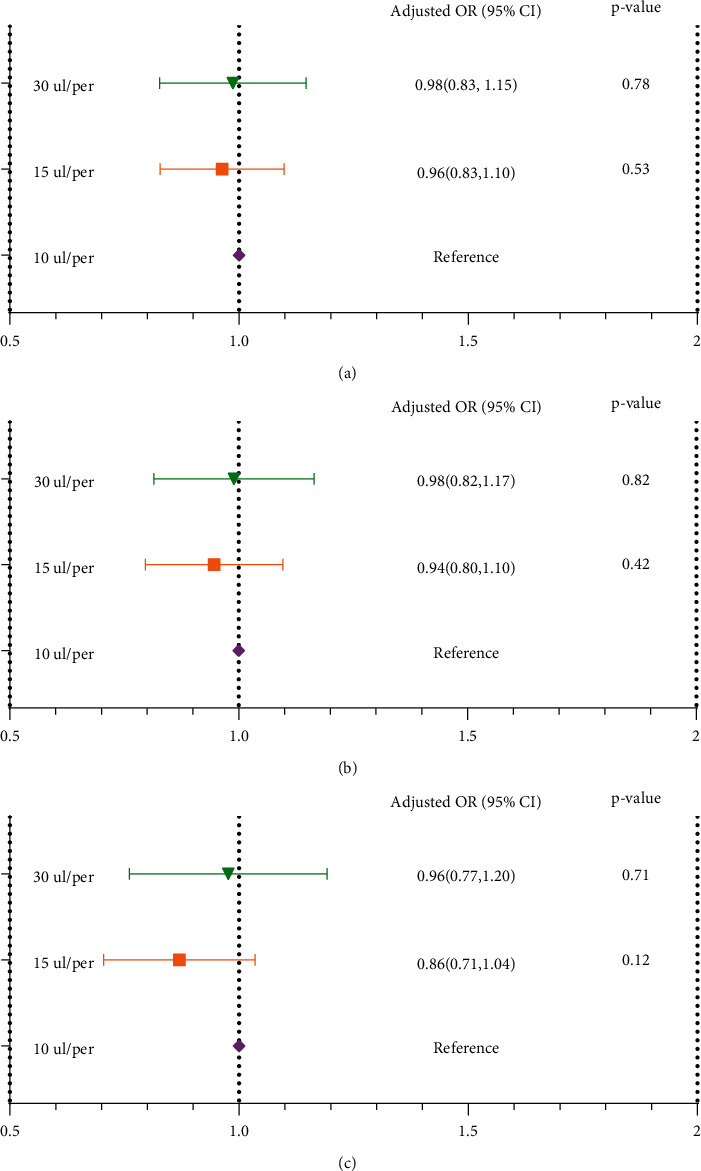
Relationship between embryo density and quality of day-3 embryos in different models. The analysis was performed by GEE (generalized estimate equation) model, subject ID = patient unique medical record number (independence). (a) Crude model: no factor was adjusted; (b) Model I: the number of retrieved oocytes, incubator COOK8, culture medium, and primary infertility were adjusted; (c) Model II: maternal age, paternal age, maternal BMI, type of infertility, type of insemination, COS protocol, AMH, the number of retrieved oocytes, length of stimulation, antral follicles, incubator, and culture medium were adjusted.

As shown in Figure 4, there was no adjustment in the crude model, and the embryo density had an insignificant effect on the embryos' quality (group 30 ul/embryo: *P*=0.78, group 15 ul/embryo: *P*=0.53, group 10 ul/embryo: reference). Infertility type, incubator, culture medium, and number of retrieved oocytes (significant risk factors in the univariate analysis) were adjusted in Model I, and embryo density still had an insignificant effect (group 30 ul/embryo: *P*=0.82, group 15 ul/embryo: *P*=0.42, group 10 ul/embryo: reference). All confounders were adjusted in Model II, and the embryo density still had an insignificant effect (group 30 ul/embryo: *P*=0.71, group 2: *P*=0.12, group 10 ul/embryo: reference).

**Figure 5:**
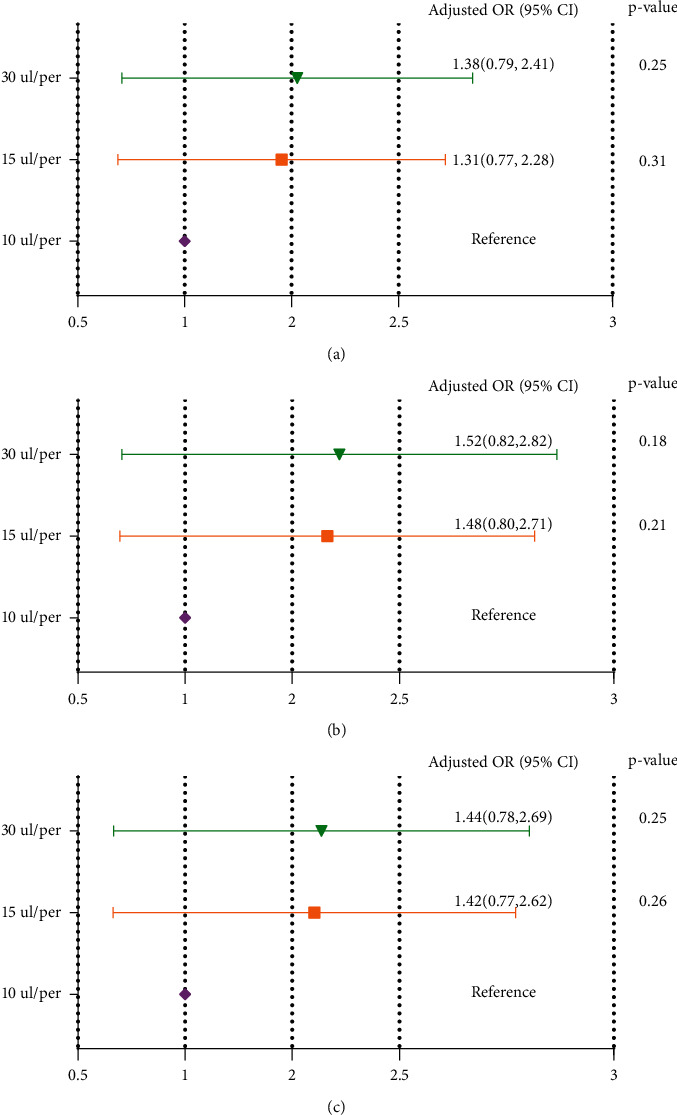
Relationship between embryo density and clinical results in different models. The analysis was performed by GEE (generalized estimate equation) model, subject ID = patient unique medical record number (independence). (a) Crude model: no factor was adjusted; (b) Model I: maternal age, paternal age, AMH, and length of stimulation were adjusted; (c) Model II: maternal age, paternal age, maternal BMI, type of infertility, type of insemination, COS protocol, AMH, the number of retrieved oocytes, length of stimulation, antral follicles, incubator, and culture medium were adjusted.

As shown in Figure 5, there was no adjustment in the crude model, and the embryo density had an insignificant effect on the clinical results (group 30 ul/embryo: *P*=0.25, group 15 ul/embryo: *P*=0.31, group 10 ul/embryo: reference). Maternal age, paternal age, AMH, and length of stimulation (significant risk factors in the univariate analysis), and embryo density still had an insignificant effect (group 30 ul/embryo: *P*=0.18, group 15 ul/embryo: *P*=0.21, group 10 ul/embryo: reference) were adjusted in Model I. All confounders were adjusted in Model II, and the embryo density still did not have an impact on the clinical results (group 30 ul/embryo: *P*=0.25, group 15 ul/embryo: *P*=0.26, group 10 ul/embryo: reference).

## 4. Discussion

In this study, the relationship between embryo density and day-3 embryo-developmental outcomes was investigated using the logistic regression in the GEE analysis. So far, this was the largest study in which researchers evaluated the association between embryo density and developmental outcomes. It is also the first study in which the confounders were adjusted to analyze the relationship between the embryo density and developmental outcome with a generalized, estimate equation. Our results showed that in the 30 *μ*l microdrop of the cleavage medium, day-3 embryo-developmental outcomes (group culture with a combination of two or three embryos) were similarly compared to the individual culture of one embryo after hypoxia culture for 48 h.

When the embryos, cultured in the 30 *μ*l/embryo and 10 *μ*l/embryo groups, were present in the individual and group cultures, the benefits were analyzed and compared between the group and individual cultures. No benefit was evident, regardless of confounder adjustment, which was consistent with Spyropoulos et al. [13]. However, other studies had different conclusions. Ebner et al. [2] reported that the compaction and blastulation were significantly higher, and the blastocyst quality was better when the embryos were cultured in groups as compared to being individually cultured in 30 *μ*l microdrops. The researchers also found that the group culture had the same top-quality of day-3 embryos as the individual culture. Therefore, we hypothesized that different endpoints largely contributed to the inconsistency, and group-culture benefits might be more obvious only when the zygotes were cultivated in vitro for a relatively longer period, such as 5 days (the in vitro environment had a longer contact with the embryos). Culture-volume variation may be another factor that contributed to the differences. Studies in which researchers used large culture volumes (>500 *μ*l) demonstrated that group culture was superior to individual culture. For example, Almagor et al. [14] indicated that after adjusting for maternal age and treatment cycle numbers, the communal growth of embryos significantly improved pregnancy rate as compared to the individual culture in a drop size of 700 *μ*l. Moessner et al. [15] found that the cleavage rates, embryo scores, and mean-cell number of day-2 embryos were significantly enhanced by the group culture, as compared to the individual culture in a drop size of 1000 *μ*l. Therefore, we assumed that only microdrops can reveal the group culture's benefits (from paracrine factors) among embryos in a large volume [16]. Furthermore, we cultivated embryos in 5% oxygen (although the embryos were cultivated in 20% oxygen of the previous two studies); we found that variable, oxygen concentration may result in the inconsistency, and oxygen concentration was reported to affect human embryos' development [17].

Our study was also complementary to previous studies. Lehner et al. [1] studied the optimal, embryo density for group culture and found that cultures with five to six embryos in a culture volume of 25 *μ*l (4.2–5.0 *μ*l/embryo) had a higher proportion of day-3 good-quality embryos when compared to cultures of two to four (6.3–12.5 *μ*l/embryo) and seven to nine (2.8–3.6 *μ*l/embryo) embryos that were together in G1-PLUS media. Under an embryo density of 10 *μ*l/embryo, we had the same proportion of day-3 good-quality embryos as the Lehner et al.'s study. They aimed to find the optimal, embryo density that was above 10 *μ*l/embryo, while we studied different embryo densities at 10 *μ*l/embryo. Besides, the effect of embryo density may be different among species. Hoelker et al. [18] compared two embryo densities (i.e., 31 *μ*l/embryo vs. 10 *μ*l/embryo) for bovine embryos that were cultivated in a fixed microdrop size of 500 *μ*l, and the results have shown that 10 *μ*l/embryo represented the optimal, embryo density, because the embryos cultured in the 31 *μ*l/embryo group reached the blastocyst stage at a significantly lower rate than the zygotes cultured in the 10 *μ*l/embryo group (22.2% vs. 30.3%). Vutyavanich et al. [6] compared four embryo densities (i.e., 10, 2, 1, and 0.67 *μ*l/embryo, respectively) for mouse embryos cultivated in a fixed microdrop size of 10 *μ*l, and their results showed that the 0.67 *μ*l/embryo represented the optimal, embryo density based on the endpoint of the mean number of ICM cells. Sananmuang et al. [19] compared three embryo densities (i.e., 2.5–2, 6.25–5, and 12.5–10 *μ*l/embryo, respectively) for eight to ten cat embryos cultivated in varied microdrop sizes of 20, 50, and 100 *μ*l, and their results showed that 2.5–2 *μ*l/embryo represented no optimal embryo density based on the endpoint of the blastocyst formation rate. Besides, the optimal, embryo density for the same species varied according to different, culture conditions (e.g., the composition of culture medium, oxygen concentration, and Petri dish type). Kelly et al. [20] concluded that under 20% oxygen, a high embryo density decreased the proportion of mouse embryos that developed to blastocyst stage on day 4 compared with low embryo density; on the contrary, a high embryo density increased the total number of blastocyst cells and trophoblast cells (under 5% oxygen), compared with low embryo density. Hoelker et al. [18] found that the well dish significantly improved the bovine embryo-developmental potential in the blastocyst stage as compared to the group culture (in the conventional dish) under the same embryo density (31 *μ*l/embryo). Therefore, further in-depth studies are still needed to investigate different embryo densities for group culture under the culture conditions similar to our study.

Importantly, we came to different conclusions as compared to previous studies regarding the effect of individual culture, group culture, and different, embryo-density culture on embryo-developmental outcomes. In this real-world study, all 10941 day-3 embryos were cultivated within a year from our center, which revealed the insignificant impact of embryo density (in the clinical practice). Although our study provides evidence on the adjusting of confounding factors and the uses of the GEE model in the analysis of embryo-developmental outcome (for future studies in the IVF laboratory), there were limitations in this study. First, there was no developmental-outcome endpoint for the blastocysts; therefore, we do not have suggestions for laboratories that cultivate all their zygotes to blastocysts. Second, although we used the GEE model in our methods, not all patients had their embryos cultivated in all three embryo-density groups, which might have decreased the power of our results. Finally, for the limitations of a retrospective study, the number of embryos in each embryo-density group was significantly unequal, which might have reduced the results' significance.

In conclusion, our results showed that in the 30 *μ*l microdrop, embryos cultured with an embryo density of 15, 10, and 30 *μ*l/embryo from zygotes to day 3 resulted in similar, developmental outcomes. Embryo density had no impact on day-3 embryo development.

## Figures and Tables

**Table 1:** Demographic and treatment cycle characteristics of all participants (*n* = 1568).

Characteristics	All
Maternal age (years, median, Q1–Q3)	33 (30–36)
Paternal age (years, median, Q1–Q3)	35 (31–38)
Maternal BMI (kg/m^2^)	22.3 (20.3–25.4)
Antral follicles (number, median, Q1–Q3)	9 (5–14)
AMH (ng/ml, median, Q1–Q3)	2.88 (1.50–5.23)
Primary infertility^a^, *n* (%)	7781 (49.7)
Length of stimulation (days, median, Q1–Q3)	10 (8–11)
Retrieved oocytes (number, median, Q1–Q3)	10 (5–15)
Long and antagonist protocol^b^, *n* (%)	1221 (77.9)
Conventional IVF^c^, *n* (%)	1044 (66.6)

AMH: anti-Müllerian hormone; IVF: in vitro fertilization and embryo transfer. ^a^Proportion of primary infertility. ^b^Proportion of long and antagonist protocol. ^c^Proportion of insemination by conventional IVF.

**Table 2:** Cleaving speed, quality, and clinical results of day-3 embryos among different embryo density groups (*n* = 10941 for embryos cleaved on day 3; *N*_1_ = 832 for embryos transferred).

Variables	Embryo density (ul/embryo)			*P* value
	30	15	10	
No. of embryos cleaved on day 3, *N*	3064	5695	2182	
7–10 cell embryos, *n* (%)^a^	1754 (57.2)	197 (56.1)	1272 (58.3)	0.73
MGE, *n* (%)^b^	613 (20)	1158 (20.3)	437 (20)	0.85
No. of embryos transferred on day 3 in the fresh cycle, *N*_1_	430	348	54	
Implanted embryos, *n* (%)^c^	162 (37.7)	129 (37.1)	15 (27.8)	0.36

MGE: morphological good embryo. ^a^Proportion of embryos with 7–10 blastomeres on day 3. ^b^Proportion of MGE on day 3. ^c^Proportion of successfully implanted embryos on day 3.

**Table 3:** Univariate analysis for cleaving speed, quality, and clinical results of day-3 embryos ^*∗*^.

Variables	7–10 cell embryos^a^		MGE^b^		Implanted embryos^c^	
	EXP (OR) 95% CI	*P* value	EXP (OR) 95% CI	*P* value	EXP (OR) 95% CI	*P* value
Embryo density (ul/embryo)						
30	1.04 (0.91, 1.19)	0.55	0.98 (0.83, 1.15)	0.78	1.38 (0.79, 0.41)	0.25
15	1.09 (0.97, 1.22)	0.15	0.96 (0.83, 1.10)	0.53	1.31 (0.77, 2.28)	0.31
10	Reference		Reference		Reference	
Maternal age, *y*	0.99 (0.98, 1.01)	0.26	0.99 (0.98, 1.01)	0.22	0.93 (0.90, 0.97)	<0.01
Paternal age, *y*	1.00 (0.99, 1.01)	0.501	0.99 (0.98, 1.00)	0.20	0.94 (0.91, 0.97)	<0.01
Maternal BMI, kg/m^2^	1.00 (0.99, 1.01)	0.617	0.99 (0.97, 1.00)	0.14	1.04 (0.99, 1.08)	0.098
Type of infertility						
Primary	1.11 (0.99, 1.23)	0.06	1.17 (1.03, 1.34)	0.02	1.32 (0.95, 1.85)	0.099
Secondary	Reference		Reference		Reference	
AMH	1.02 (1.00, 1.03)	0.04	1.01 (0.99, 1.03)	0.401	1.08 (1.01, 1.16)	0.04
Antral follicles	1.00 (0.99, 1.01)	0.49	1.00 (0.99, 1.01)	0.84	1.03 (0.96, 1.10)	0.43
COS protocol						
Long and antagonist	1.01 (0.88, 1.17)	0.89	0.91 (0.76, 1.09)	0.33	1.35 (0.79, 2.29)	0.27
Other protocols	Reference		Reference		Reference	
Length of stimulation	1.01 (0.99, 1.03)	0.45	1.01 (0.98, 1.03)	0.75	1.08 (1.00, 1.16)	0.047
No. of retrieved oocytes	1.00 (1.00, 1.03)	0.06	1.01 (1.00, 1.02)	0.09	1.01 (0.97, 1.05)	0.58
Type of insemination						
IVF	0.97 (0.87, 1.07)	0.53	0.94 (0.83, 1.07)	0.36	1.17 (0.83, 1.64)	0.38
ICSI	Reference		Reference		Reference	
Incubator						
COOKA	1.07 (0.88, 1.30)	0.52	1.11 (0.86, 1.44)	0.42	1.03 (0.54, 1.96)	0.94
COOK8	1.15 (0.94, 1.41)	0.16	1.35 (1.04, 1.75)	0.03	0.98 (0.51, 1.88)	0.94
COOK7	1.12 (0.88, 1.41)	0.36	1.07 (0.80, 1.42)	0.67	1.35 (0.60, 3.03)	0.46
COOK6	0.96 (0.79, 1.16)	0.66	1.06 (0.84, 1.4)	0.62	1.46 (0.80, 2.67)	0.22
COOK5	1.02 (0.85, 1.24)	0.81	1.08 (0.85, 1.4)	0.53	1.05 (0.56, 1.99)	0.88
COOK3	1.07 (0.88, 1.30)	0.50	1.20 (0.93, 1.5)	0.16	0.73 (0.38, 1.40)	0.34
COOK2	0.98 (0.81, 1.2)	0.87	1.14 (0.90, 1.5)	0.28	0.83 (0.44, 1.57)	0.57
COOK1	Reference		Reference		Reference	
Culture medium						
Vitrolife	1.10 (0.98, 1.22)	0.10	1.19 (1.04, 1.37)	0.02	1.36 (0.96, 1.93)	0.09
Cook	Reference		Reference		Reference	

BMI: body mass index; AMH: anti-Müllerian hormone; COS: controlled ovarian stimulation; IVF: in vitro fertilization and embryo transfer; ICSI: intracytoplasmic sperm injection; MGE: morphological good embryo. ^a^Whether the embryo cleaved to 7–10 cells on day 3. ^b^Whether the embryo was a MGE on day 3. ^c^Whether the transferred embryo was successfully implanted. ^*∗*^Analysis done by generalized estimate equation (GEE) model, subject ID = patient unique medical record number (independence).

## Data Availability

The datasets that were used or analyzed during the current study are available from the corresponding authors on reasonable request.
